# Estimating geographical spread of Streptococcus pneumoniae within Israel using genomic data

**DOI:** 10.1099/mgen.0.001262

**Published:** 2024-06-24

**Authors:** Hsueh-Chien Raymond Cheng, Sophie Belman, Henrik Salje, Ron Dagan, Stephen D. Bentley

**Affiliations:** 1 The Wellcome Trust Sanger Institute, Wellcome Trust Genome Campus, Hinxton, Cambridge, CB10 1SA, UK; 2 Global Health Resilience, Earth Sciences Department, Barcelona Supercomputing Center, Barcelona, Spain; 3 Department of Genetics, University of Cambridge, Cambridge CB2 3EH, UK; 4 Gunzburger Chair for Study of Infectious Diseases, The Shraga Segal Department of Microbiology, Immunology and Genetics, Faculty of Health Sciences, Ben-Gurion University of the Negev, Beer-Sheva, Israel

**Keywords:** pneumococcal genomics, pneumococcal spread, relative risk ratio

## Abstract

Understanding how pathogens spread across geographical space is fundamental for control measures such as vaccination. *Streptococcus pneumoniae* (the pneumococcus) is a respiratory bacterium responsible for a large proportion of infectious disease morbidity and mortality globally. Even in the post-vaccination era, the rates of invasive pneumococcal disease (IPD) remain stable in most countries, including Israel. To understand the geographical spread of the pneumococcus in Israel, we analysed 1174 pneumococcal genomes from patients with IPD across multiple regions. We included the evolutionary distance between pairs of isolates inferred using whole-genome data within a relative risk (RR) ratio framework to capture the geographical structure of *S. pneumoniae*. While we could not find geographical structure at the overall lineage level, the extra granularity provided by whole-genome sequence data showed that it takes approximately 5 years for invasive pneumococcal isolates to become fully mixed across the country.

This article contains data hosted by Microreact.

**Impact Statement**

We adapted a relative risk framework to quantify the spread of the pneumococcus while considering genomic and spatiotemporal information. This framework is written into a R package, *rrspread*, allowing users to designate relatedness and location features of pairs of isolates for the comparison in risk of spread. We highlighted that with the aid of genomic data, we have increased resolution to identify geographical structure and quantify the spread of the pneumococcus.

## Data Summary

Whole-genome sequences are deposited at the European Nucleotide Archive (ENA) and accession numbers are available with the complete metadata of this study. A phylogenetic snapshot of pneumococcal isolates from Israel is available at https://microreact.org/project/1yYH4qFbqx4tzAFCRNLNTQ-israelrelativerisk. The authors confirm that all supporting data, code and protocols have been provided within the article or through supplementary data files.

## Introduction

Quantifying migration patterns of pathogens has long been a central focus in epidemiology studies due to the implications of geographical disease spread for the effectiveness of prevention and mitigation strategies. Strategies to prevent and cure viral and bacterial diseases should be designed based on the compositions of strains in the circulating populations. Quantitative methods to understand the patterns and rates of spread across geographical space are the first step to understanding the disease dynamics and the underlying interactions among bacterial strains. Pathogen genomic data can provide high-resolution insight into the relatedness of pathogens across continuous distances and between locations [1,2].

*Streptococcus pneumoniae* (the pneumococcus) is an opportunistic human obligate bacterium that commonly resides in the nasopharynx, and carriage is a prerequisite for the progression to disease [3]. It usually resides asymptomatically or causes local infection such as otitis media, sinusitis, and pneumonia. It can, however, invade normally sterile sites to cause severe disease, such as bacteraemia, meningitis, and sepsis, which are referred to as invasive pneumococcal diseases (IPDs). * S. pneumoniae* has long been a leading bacterial cause of morbidity and mortality, with an estimated ~294, 000 annual deaths in children under 5 years of age globally [4,5].

In Israel, the pneumococcal conjugate vaccines (PCVs), PCV7 and PCV13, which targeted 7 and 13 different serotypes, were introduced in July 2009 and November 2010, respectively [6,7]. PCVs, where multiple polysaccharide capsule structures are conjugated to a protein carrier, have been broadly utilised since the early 2000s to reduce the spread of pneumococcal disease. Concerningly, despite being targeted in the vaccine, serotypes 3, 14, and 19A are still the most common serotypes, with no significant decline throughout the vaccination period [7]. Serotype replacement after vaccine implementation is a phenomenon in which non-vaccine types (NVTs) increase in frequency following reduction in vaccine types (VTs) [8]. The increase of NVT frequency is either a result of serotype switching via bacterial recombination at its *cps* locus [9], or the expansion of pre-existing NVT isolates [10]. In Israel, multiple non-vaccine targeted serotypes, including serotypes 2, 8, 12F, and 24F, became more prevalent following the introduction of PCV [7,11,13]. Despite the increasing prevalence of these serotypes, the rates of IPDs have significantly declined in children <5 years by 67 % following PCV introduction [7].

Pneumococcus possesses high genetic diversity due to mutation events, frequent horizontal gene transfer, and selection pressure from diverse human hosts [14,16]. The polysaccharide capsule that surrounds the bacterial cell is essential for evading the human immune system and is the basis for the serotyping classification scheme [17,18]. The pneumococcus comprises more than 100 antigenically distinct serotypes, a subset of which are the target of current vaccines [8,10,19]. Global pneumococcal sequence clusters (GPSCs) have been established as an international genomic definition of pneumococcal lineages and are classified using PopPUNK, a tool for genome-wide variable-length k-mer comparison of both core and accessory genes [1,20, 21]. To date, the pneumococcus comprises >900 GPSCs.

In this work, we aim to quantify pneumococcal spread across Israel. Israel is 424 km from north to south and at its widest point is 114 km from east to west. We first aim to investigate whether there is geographical structure for the pneumococcal population in Israel. We can address this aim using both GPSC and evolutionary divergence but with different resolution, where evolutionary divergence shows higher resolution, as indicated in our results. Once we identify that there is geographical structure, we would like to ask how long it takes for invasive pneumococcal isolates to become homogeneously mixed across Israel, which is our second and ultimate aim, namely quantifying the spread of pneumococcus in Israel. We utilise a relative risk (RR) framework previously implemented to understand pneumococcal spread in South Africa, and to interrogate the spread of a multidrug-resistant emergent strain in France [2,22]. This relative risk framework is written into an R package, *rrspread* (https://github.com/hsuehchien66/rrspread_v2), to facilitate the quantification of disease spread in different geographical settings. Ultimately, we are working towards contextualising pneumococcal migration in Israel and amongst other countries to infer possible drivers of spread.

## Methods

### Study design

**Fig. 1.**
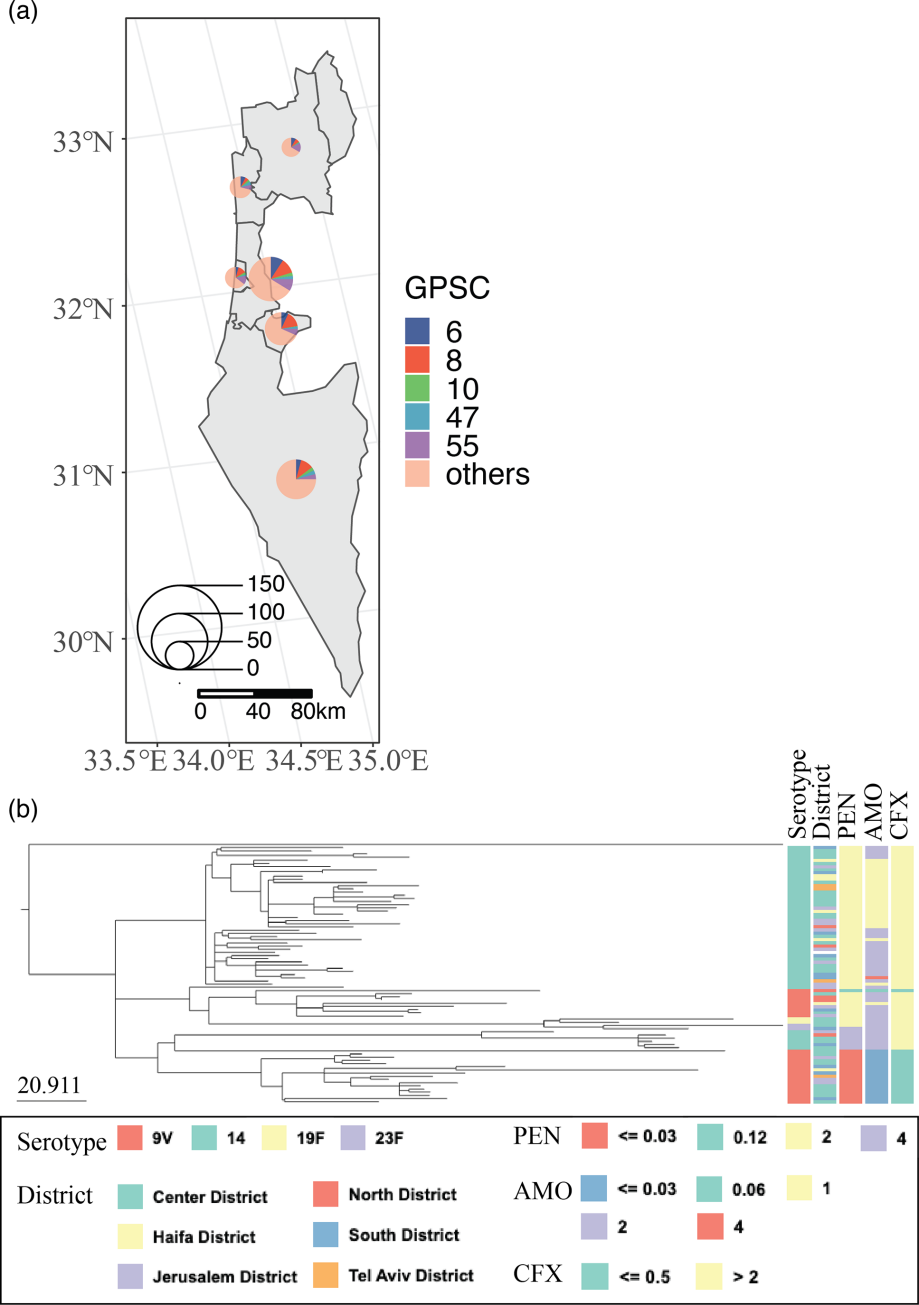
The geographical distribution of the pneumococcal isolates in Israel included in the study. (**a**) The geographical distribution of collected pneumococcal isolates in Israel in the study. The pie charts indicate the proportion of the GPSCs and their sizes indicate the number of samples in the administrative districts. (**b**) The recombination-corrected phylogenetic tree of GPSC6 isolates and their serotype, district, and AMR profiles.

We included 1174 Israeli IPD isolates from the Global Pneumococcal Sequencing Project (https://www.pneumogen.net/gps/project_outline.html), collected between 2005 and 2014. Epidemiological information included sample source, collection sites, isolation month and year, host age and gender, vaccination status, and clinical manifestation. These isolates came from six different administrative districts: Center District, Haifa District, Jerusalem District, North District, South District, and Tel Aviv District (Fig. 1 and Table S1, available in the online version of this article). Isolates were sampled from a typically sterile site, either blood or cerebrospinal fluid. Each isolate was sequenced by Illumina paired-end whole-genome sequencing and *de novo* assembled via an automated bacterial genome assembly pipeline [23].

### Data inclusion criteria for divergence time analysis

We included GPSCs with a minimum of 20 genomes and a linear correlation between the sampling date and root-to-tip distance in the inference of divergence time (Figs S7 and S8). We used GPSC throughout the analysis as the definition of pneumococcal lineages but note that this approach can be applicable to any pathogen where there is lineage information available. We refer to these as ‘dominant GPSCs’. The dominant GPSCs comprise GPSC6 (*n*=81), GPSC8 (*n*=130), GPSC10 (*n*=27), GPSC47 (*n*=32), and GPSC55 (*n*=91).

Microreact software data display for dominant GPSCs:

- GPSC6: https://microreact.org/project/mCJqo6q7EgDHvgJrCgJpCf-israelgpsc6;
- GPSC8: https://microreact.org/project/jc294xukujvPgN93JmAMqK-gpsc8israelmicroreact;
- GPSC10: https://microreact.org/project/2pzbKrxowreLkiyuxXXFtF-gpsc10israelmicroreact;
- GPSC47: https://microreact.org/project/h4S84wHJ213Uy8Hnnn9Jdd-gpsc47israelmicroreact;
- GPSC55: https://microreact.org/project/4ptHsdUapBX798mTyMKU3M-gpsc55israelmicroreact.

### *In silico* serotyping, lineage classification, and antimicrobial resistance detection

The serotype of each isolate was derived from the genome using SeroBA [24]. GPSC lineage was designated by PopPUNK [20]. Antimicrobial resistance (AMR) for penicillin (PEN), amoxicillin (AMO), meropenem (MER), cefotaxime (TAX), ceftriaxone (CFT), and cefuroxime (CFX) was predicted by the CDC pneumococcal typing pipeline database and algorithm [25,26].

### Generating time-resolved phylogeny

To investigate the relationship between the spread of disease and evolutionary divergence, we calculated divergence time between isolates. For each GPSC, we first created a reference-guided GPSC-specific reference genome by using ABACAS [27] to order the contigs relative to strain ATCC 700669/Spain 23 F-1 (EMBL accession: FM211187). Any contigs that did not align were concatenated to the end. For isolates within the same GPSC, we aligned the isolates to the corresponding GPSC-specific reference by BWA alignment and variants were called by samtools [28,29]. We masked recombinatory sites using Gubbins with RAxML and a general time-reversible (GTR) model [30,31]. To rescale the branch lengths to time we used BactDating with the additive uncorrelated relaxed clock (ARC) model [32,33]. This utilises a Bayesian framework to estimate the coalescence time of each inner node.

### Relative risk analysis overview

We adapted a relative risk ratio framework to capture the geographical structure of *S. pneumoniae* [22]. The aim of this framework is to investigate whether there is geographical structure for the pneumococcal population in Israel. This means that isolates that are more similar genetically will be found closer together geographically than those that are less similar. We then leverage time-resolved phylogenies to quantify the time that it takes for the pneumococcus to spread across geographical space.

Conceptually, the relative risk ratio allows us to quantify the diversity of pneumococcal lineages across geographical space. We sum the number of isolates that are in the same GPSC and observed in the same location and compare that to the number of isolates that are in the same GPSC and observed in the different locations. Within this framework we are able to normalise by the number of isolates that are not in the same GPSC, allowing us to adjust for population variation and apply this framework across geographical locations. When this ratio is larger than 1, it suggests that the isolates in close genetic relatedness tend to be observed in the same location, or namely, there is geographical structure of the bacterial population.

The mathematical description of the relative risk ratio framework is described by *Belman et al.* [22] and shown in the following equations. In the relative risk ratio framework, we compare the location (L), genetic relatedness (G) and collection time (T) for each pair of isolates *i* and *j*. When comparing genetic relatedness, we use either lineage or pairwise divergence time information. When using lineage information, *G*_*i*_=*G*_*j*_ if *i* and *j* are in the same lineage. GPSC is used as lineage definition throughout the whole analysis. When using pairwise divergence time, *G*_*i*_=*G*_*j*_ if *i* and *j* are within the designated pairwise divergence time window. When comparing the location, we use either district or pairwise spatial distance information. When using district information, *L*_*i*_=*L*_*j*_ if *i* and *j* are in the same district. If we use pairwise spatial distance information, *L*_*i*_=*L*_*j*_ if *i* and *j* are within the designated pairwise spatial distance window. When comparing the collection time, we use sample collection time information. T_*i*_=T_*j*_ if *i* and *j* are in the same collection time period. $L_{ref_{i}}=L_{ref_{j}}$ in Equations 3 and 4 indicates that the pair of isolates are located in different districts when using district information, or they are distant pairs that are within a reference distance window. In the relative risk ratio (Equation 5), *a* indicates the number of pairs of isolates that are in the same location, same collection time period and same GPSC or within short divergence time (Equation 1); *b* indicates the number of pairs of isolates that are in the same location and same collection time period regardless of their genetic relatedness (Equation 2); *c* indicates the number of pairs of isolates that are in the different locations, same collection time period and same GPSC or within short divergence time (Equation 3); *d* indicates the number of pairs of isolates that are in the different locations and same collection time period regardless of their genetic relatedness (Equation 4). The relative risk ratio is calculated in Equation 5. We performed 20 bootstrap iterations to generate the confidence interval. For each iteration, we first randomly selected 70 isolates in each district as every district includes at least 70 isolates. We then performed resampling with replacement for all the selected isolates from all districts. With these resampled isolates, we calculate the relative risk ratio.

In practice, there are four steps involved in the *rrspread* package: (1) perform bootstrapping to resample the isolates from different locations, (2) generate pairwise matrices to include pairwise information of location, genetic relatedness and collection time, respectively, (3) multiply the matrices to count the number of isolate pairs that meet the conditions, and (4) calculate the relative risk ratio. We used this framework to perform the following analysis:

### Quantifying geographical structure at lineage level

To understand whether distinct GPSCs are circulating in different regions of Israel, we first compared the relative risk of observing isolates from the same GPSC and time period in the same versus different districts. All 1174 Israeli isolates were included in this analysis. We compared every pair of isolates that were the same GPSC (*G*_*i*_=*G*_*j*_), collected within the same district (*L*_*i*_=*L*_*j*_) to the number of all pairs within the same district (*L*_*i*_=*L*_*j*_) in the numerator. The denominator included the number of pairs collected in different districts () which were the same GPSC, over the all pairs collected in different districts irrespective of GPSC ($L_{ref_{i}}=L_{ref_{j}}$).

In addition to using the district information of each individual isolate, we used the coordinates of the hospitals from which it was identified to explore the spatial dependence in GPSC across different spatial windows. To do this, we conducted a rolling window analysis where we compared the relative risk of observing the same GPSC in a designated distance window to a reference window. For the selection of reference windows, we performed sensitivity analysis on different reference windows, including all pairs of isolates, pairs >50 km, pairs >80 km, and pairs >100 km apart.

### Quantify the homogenisation time of *S. pneumoniae* in Israel

To elucidate how long it takes for *S. pneumoniae* to become homogeneously mixed across Israel, we investigated the relationship between divergence time and geographical distance. Dominant GPSCs (see the ‘Data inclusion criteria for divergence time analysis’ section above) were selected to perform the following analysis. We incorporated phylogenetic uncertainty using the BactDating posterior. We then compared the relative risk of observing the isolates within a designated divergence time in the same and different districts by using rolling windows for designated divergence time. In the same framework as quantifying geographical structure at lineage level **,** we replaced the pairwise lineage matrix with pairwise divergence time matrix. We also conducted the same analysis by distance where we compared the relative risk of observing the isolates within a designated divergence time and within 30 km to different reference windows (see ‘Quantifying geographical structure at lineage level’) by using rolling windows for designated divergence time.

## Results

The 1174 pneumococcal genomes included in this study comprise 103 GPSCs and 51 serotypes across Israel (Fig. 1a, Tables 1 and S1). The geolocated distribution of *S. pneumoniae* from the study was recorded to the hospital level. Approximately 75 % of the collection were from children aged under 5 years old. The lineage-specific propensity for carrying multiple serotypes was independent from sample size, as the most prevalent lineage, GPSC8, is only linked to serotype 5, while the less prevalent lineage, GPSC10, is linked to seven different serotypes (Table 1). The dominant GPSCs of focus for this study include five GPSCs: GPSC6, GPSC8, GPSC10, GPSC47 and GPSC55 (Figs 1 and S1 and Table 1), and the pairwise distance distribution is shown in Figs S2 and S3. The mean pairwise distance across all pairs of isolates was 62 km (Q1=28, Q3=88, min=0, max=272). The number of isolates collected from each district per year is shown in Table 2.

**Table 1.** The epidemiological information for *S. pneumoniae* isolates in Israel

**GPSC**	***n***	**No. of serotype**	**No. of VT isolates**	**No. of NVT isolates**
All	1174	51	771	403
GPSC8	130	1	130	0
GPSC55	91	1	0	91
GPSC6	81	4	81	0
GPSC47	32	2	32	0
GPSC10	27	7	16	11

VT, PCV13 vaccine type; NVT, non-PCV13 vaccine type.

**Table 2.** The number of samples collected per district per year

**Year**	Center	**Haifa**	**Jerusalem**	**North**	**South**	Tel Aviv
2005	33	8	18	7	27	5
2006	45	4	29	1	43	4
2007	51	9	18	3	53	5
2008	14	4	6	1	43	1
2009	56	11	55	7	33	14
2010	35	8	25	7	35	6
2011	48	13	15	13	31	16
2012	46	13	17	15	17	14
2013	38	17	22	10	21	15
2014	25	6	10	8	10	10

### Geographical structure for *S. pneumoniae* is not found at lineage level in Israel

**Fig. 2.**
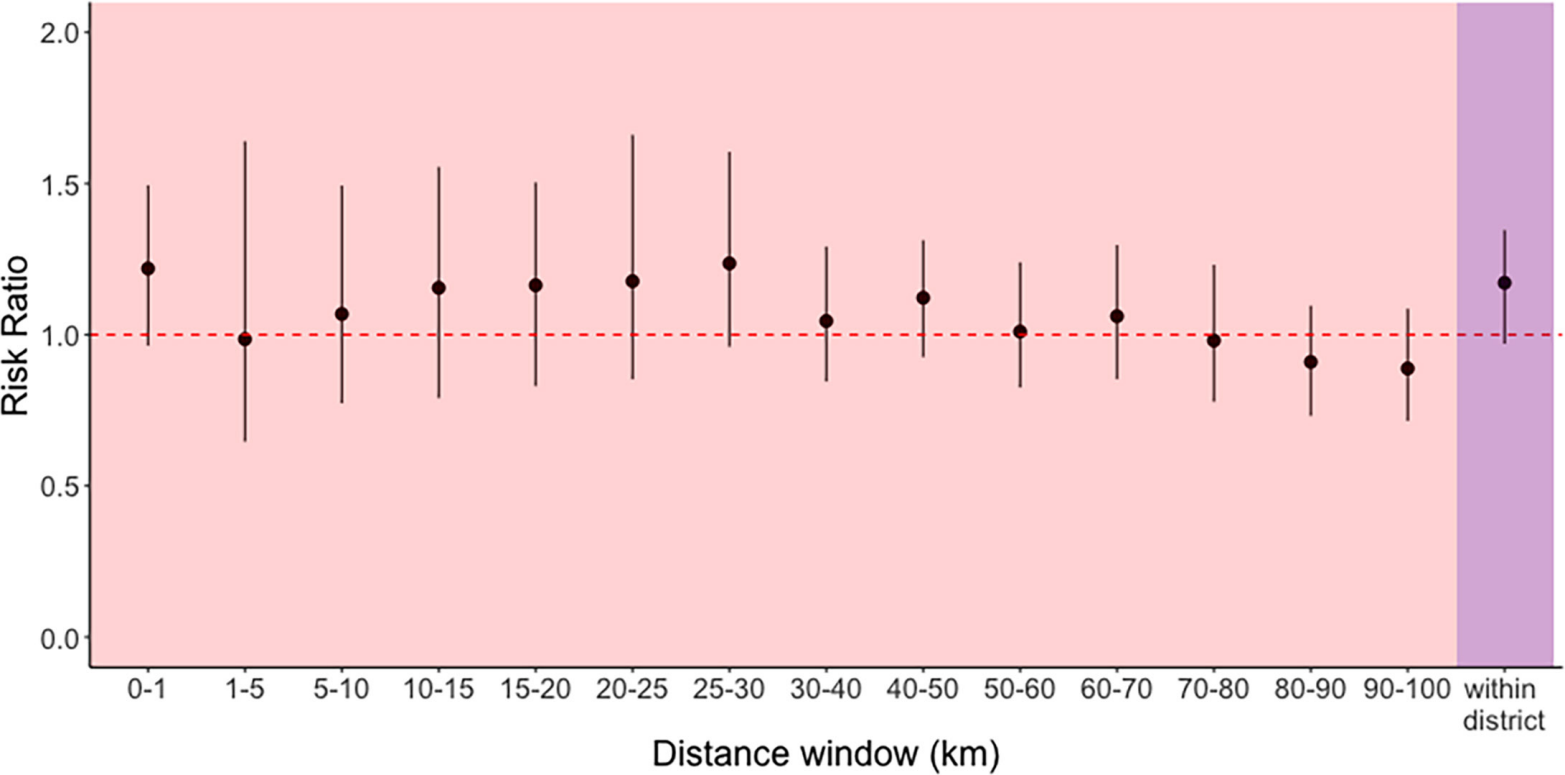
Quantifying geographical structure at lineage level. Relative risk ratio of spread at lineage level was calculated using Israeli disease isolates (*n*=1174). Purple: relative risk ratio of spread at lineage level when comparing pairs of isolates within districts to between districts. Pink: relative risk ratio of spread at lineage level when comparing pairs of isolates within the rolling distance windows to all pairs of isolates. Red dashed line highlights a relative ratio of 1, representing a significant difference in spread. The dots and lines represent 2.5, 50, and 97.5 percentiles of the confidence intervals.

To investigate the geographical structure of *S. pneumoniae*, we first focused on the spread at lineage level across Israel. We first investigated whether a higher risk of the same lineages circulating in the same districts, as compared to different districts, existed using a relative risk ratio framework (Methods). We found that there was no distinct geographical structure in the distribution of circulating lineages at the district level (RR=1.17, 95 % interval: 0.97–1.34, Fig. 2). This indicates extensive pneumococcal mixing in Israel and it is not stratified by administrative districts. To increase the resolution of geographical structure and delineate how the migration pattern changes as the spatial distance increases, we compared similarity across continuous geographical distances. We found that pairs of isolates collected within all distance windows showed no distinct geographical structure, regardless of comparing different reference windows (Figs 2 and S4). Even for pairs observed within 1 km, the relative risk of spread was not significant compared to all pairs of isolates (RR=1.22, 95 % interval: 0.96–1.49, Fig. 2). In summary, geographical structure of pneumococcus at the lineage level was not found when using administrative districts or geographical distances.

### Estimating the homogenisation time of *S. pneumoniae* in Israel

**Fig. 3.**
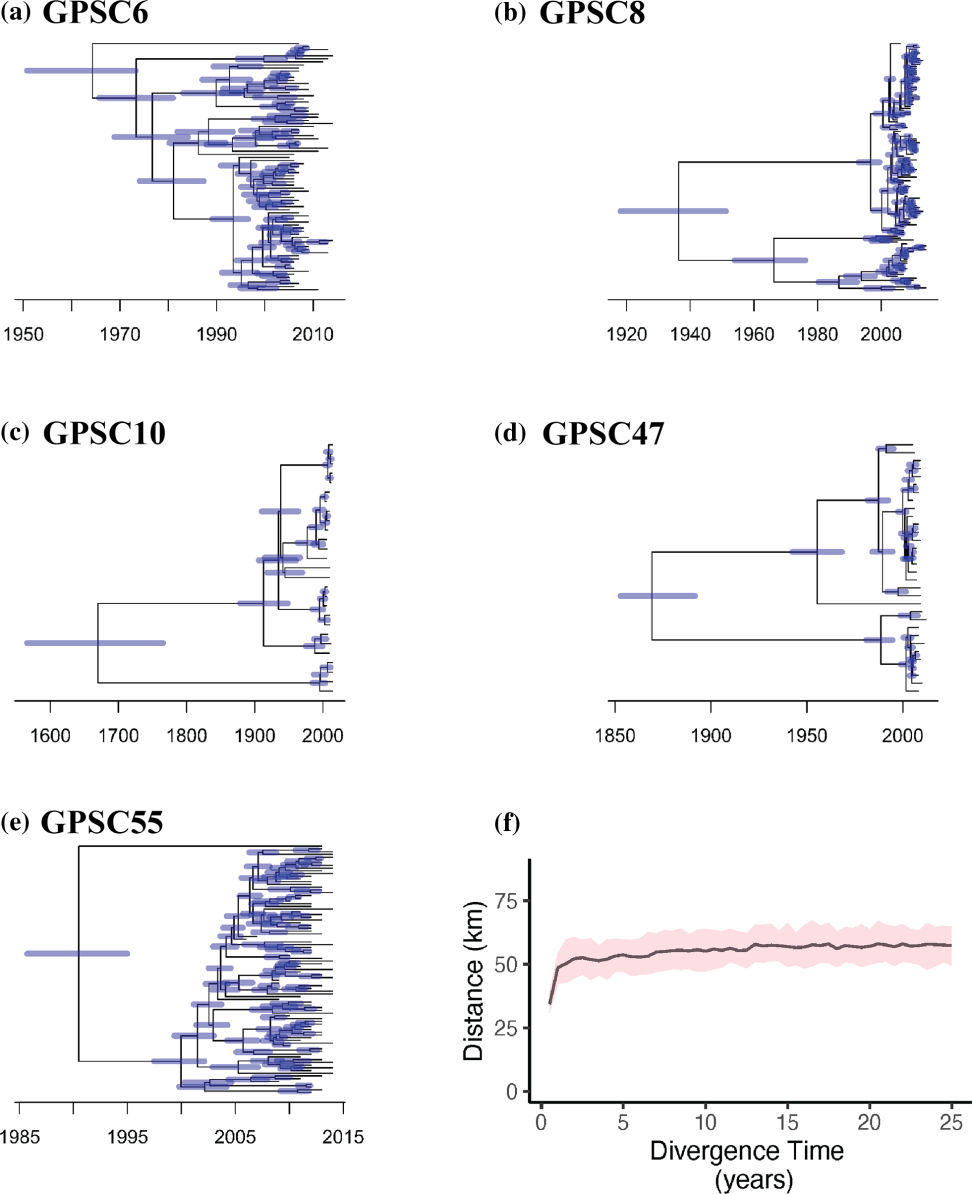
The relationship between pairwise divergence time and physical distance in Israel. (**a–e**) The dating of internal nodes with 95 % credible interval (blue) in GPSC6, GPSC8, GPSC10, GPSC47, GPSC55 phylogenies. (**f**) The relationship between pairwise cumulative divergence time and mean geographical distance with 95 % credible interval (red) across all five GPSCs in Israel.

The genetic diversity within a lineage can be very broad, with up to hundreds of years of evolutionary diversity within a GPSC. This substantial diversity can hide patterns of spread when analysing spatial structure at the lineage level. We therefore conducted an analysis where we focused on the increased granularity provided by the pairwise evolutionary divergence times between isolates within lineages. To gain correct estimates of divergence time, we only included the dominant GPSCs in the following analysis (Fig. 3a–e). The nucleotide substitution rates (substitutions per year) estimated by BactDating were inferred across these five lineages (GPSC6: µ=3.44, σ=5.60; GPSC8: µ=1.69, σ=0.74; GPSC10: µ=2.99, σ=6.77; GPSC47: µ=2.69, σ=2.85; GPSC55: µ=2.46, σ=0.47). We investigated the relationship between spatial distance and divergence time for each GPSC. We found increasing geographical distances between pairs of isolates that had diverged within 5 years. The mean distance between pairs of isolates plateaus at around 57 km (95 % CI: 51.83–60.18 at divergence time <5 years) (Fig. 3f). This is similar to the mean pairwise distance between all pairs of isolates in our dataset, irrespective of GPSC.

**Fig. 4.**
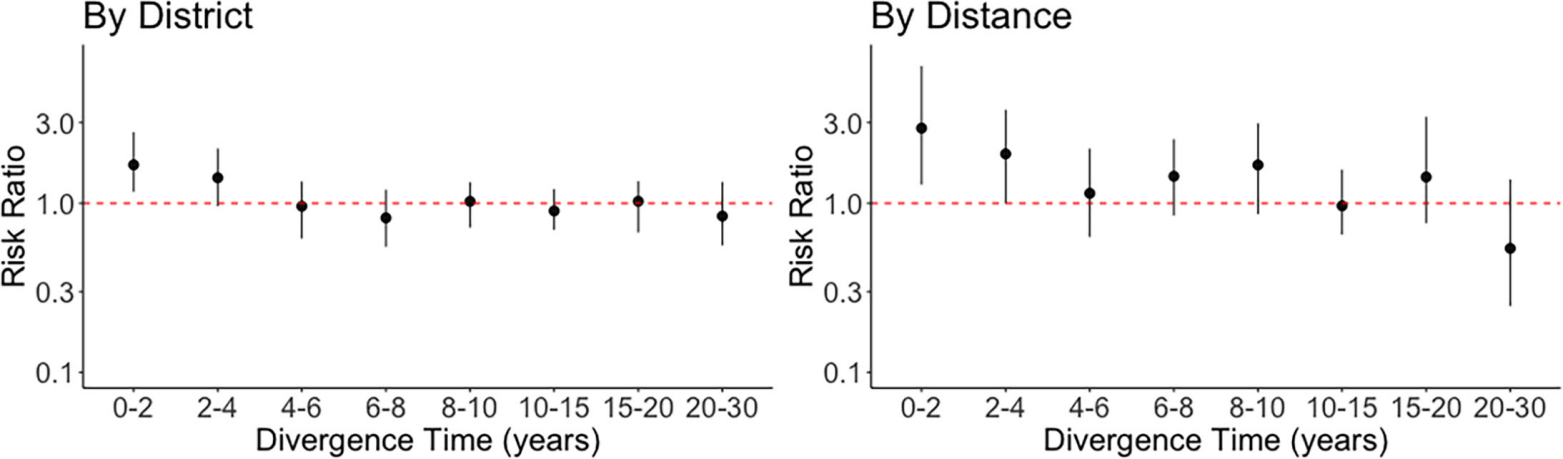
Divergence time analysis on *S. pneumoniae* migration dynamics. The relationship between pairwise divergence time and relative risk ratio was investigated using five dominant GPSCs in the study (*n*=361). The relationship between pairwise divergence time and relative risk ratio when comparing within district and between districts (left) and when comparing pairs of isolates within 30 km and pairs of isolates larger than 100 km apart (right). Red dashed line highlights a relative ratio of 1, representing a significant difference in spread. The dots and lines represent 2.5, 50, and 97.5 percentiles of the confidence intervals.

To investigate how long it takes for *S. pneumoniae* to spread across the Israel population, we conducted a rolling window analysis across divergence times to calculate the risk of spread across discrete administrative districts and across continuous distances. All results showed a rapid decrease to relative risk ratio of 1 when increasing the divergence time between pairs to 4–6 years, regardless of using administrative districts or different reference distances (Figs 4 and S6). This indicates that it takes approximately 5 years for invasive pneumococcal isolates to become fully mixed across Israel.

## Discussion

The extra granularity provided by whole-genome sequence data has allowed us to investigate the quantitative spread of the pneumococcus. In this study, using single-nucleotide variation data, we identify a positive correlation between spatial distance and evolutionary divergence time, showing that a newly introduced strain of *S. pneumoniae* would be well mixed across the country of Israel within a 5 year period. Given this rate of spread across the country, we do not observe clear geographical structure with only lineage-level resolution. This study demonstrates that stratifying pathogens at lineage level without detailed genomic information is not sufficient to detect spatial structure in the scenario of rapid spread.

The homogenisation time for pneumococcal population of 5 years in Israel shows a relatively rapid spread of pneumococcus within the country. In contrast, a previous study in South Africa was able to show geographical structure at the lineage level and estimated 50 years for a newly introduced strain to be fully mixed across the country [22]. This contrast may be due to the difference between the geographical size and the speed of human travel within each of the two countries. Geographically and demographically, Israel is approximately 50-fold smaller and 8-fold more densely populated than South Africa, in the studied period. As for internal human migration, around 17 % of the population on average migrates between different localities in Israel every year [34]. Among these Israeli internal migrants, a substantial number move long distances, specifically from urban to rural areas and from rural to urban.

Several factors have contributed to the frequent and long-distance travel and, in turn, faster spread of pneumococcus in Israel. Commonly, urban areas still provide better opportunities of education and employment that motivates travelling and migration toward urban areas, whereas the growing urbanisation in Israel has incentivised citizens to move to suburban areas due to the high cost of living in major cities and better quality of life in rural areas [34,35]. Policies from the Israeli government have also been formulated to encourage population dispersion [36]. Furthermore, the establishment of efficient transportation and high road density facilitate travelling and migration without job transfer [37,38]. High birth rate and high accessibility to day care centres might enhance the spread of pathogens within children as well as increased travels between day centres and households [39]. It has also been demonstrated that a new strain occurring in a rural municipality can spread farther within a certain period of time than from an urban municipality [22]. Together, continuous internal migration accompanied by everyday commutes provide more intricate and rapid networks for the spread of pathogens in Israel. With the availability of human travel data and transportation networks, mechanistic models can be applied to describe the variance of spread resulting from human movements [22,40].

While it is clear that the overall spread of pneumococcus in Israel is comparatively rapid, there is evidence for variation in rates of spread across GPSCs. For example, the pairwise geographical distances of isolates in GPSC6, GPSC10, and GPSC47 increases as their divergence time increases within 10 years, while this positive relationship between geographical distance and evolutionary divergence was not observed in GPSC8 and GPSC55 (Fig. S5). This observation coincides with older most recent common ancestor (MRCA) in GPSC10 and GPSC47 and more recent MRCA in GPSC8 and GPSC55. Further GPSC-wise analysis using global datasets would be helpful to investigate the relationship between the MRCA date and geographical structure. Although the small sample size in each GPSC and uncontrolled confounding factors may affect the pattern of spread, our results may be indicative of intrinsic genetic differences impacting on their ability to spread. In addition, different GPSCs have unique evolutionary histories. Specifically, GPSC55 is exclusively detected in Israel and the USA in the Global Pneumococcal Sequencing dataset (https://www.pneumogen.net/gps/project_outline.html), correlating with the frequent human migration between the two countries according to the UN migration report [41]. Recent clonal expansion, long branches between isolates, and uneven sampling times have created difficulties for using a standardised way of generating correct time-resolved phylogeny. In some GPSCs, separating these lineages into sub-clades improved divergence time inferences (Fig. S9). More accurate methods based on the genetic distance (i.e. PopPIPE: https://poppunk.readthedocs.io/en/latest/subclustering.html) could be used to divide the samples within the same GPSC lineage. Deep sequencing methods to identify co-carried isolates may also recover the unobserved spread.

Multiple key factors related to bacterial spread have not been explored explicitly. While the samples were collected from the medical service and all of them are IPD samples, we are not sure whether the carriage isolates have a similar rate of spread. However, due to carriage being a prerequisite for invasive disease, asymptomatic carriage samples are embedded in the transmission chains and the progression to disease. Our lack of asymptomatic carriage isolates does not mean that they are totally neglected. We also recognise that age-specific mixing patterns may result in some age-dependence in our results if there was significant preferential transmission between individuals of a similar age, i.e. adults tended to infect adults and children tended to infect children. Further analysis explicitly explores the spread of asymptomatic isolates and in separate age groups using the similar framework would be beneficial to fill this gap. Investigation into the geographical structure of *S. pneumoniae* isolates comprising certain vaccine types and non-vaccine types may have relevance to post-vaccination dynamics. PCV implementation is likely to have an impact on host herd immunity to the targeted serotypes, reducing their spread. However, we do not have the statistical power within this dataset to stratify the samples by vaccine type and non-vaccine type. Further relative risk analysis could include vaccine type and serotype matrices to observe whether vaccination implementation would influence risk of migration on vaccine-targeted serotypes. With accumulating genomic or serotyping data, serotype replacement could additionally be investigated by incorporating serotype matrices into the relative risk calculation. It may be valuable in future to stratify the risk of migration in different serotypes and in pre- and post-vaccination periods.

Relative risk ratio is a simple and flexible statistical framework that allows us to include useful information when quantifying the spread of the pathogen as well as allowingf us to account for the diversity of the pneumococcus while including samples from different GPSCs. Previously, researchers have been using a phylogeographical model included in beast, which requires the use of a single phylogenetic tree [42]. This method will be useful under epidemic settings when clonal expansion occurs. However, due to the diversity in pneumococcus across multiple years, it is not possible to generate a single accurate phylogenetic tree. By leveraging the divergence time between pairs we can include temporal signals in the relative risk ratio framework. Furthermore, we can account for additional variables, such as the collection time between pairs, or the age difference between those sampled, as has been done in the South African population [22]. Where sufficient data are available, other epidemiological factors, such as HIV exposure and vaccination status, can be taken into account by adding more pairwise matrices. Here, we have written functions and formatted the framework into an R package to make it flexible and easy to implement. This can be applied more broadly in different species, which will enable a better determination of how the spread of the pathogens varies geographically.

In summary, we have used genomic data to quantify the spread of invasive pneumococcal isolates in Israel. With the continued growing size of available genomic data, it may be possible to identify key genetic variants contributing to the spread of the pathogen. The rapid spread shown in this analysis could be further investigated with the information of human mobility networks, co-carriage of different lineages, duration of carriage, and the spread of carriage samples, to demonstrate possible mechanisms of pneumococcal migration affected by human mobility and microbial interactions.

## supplementary material

10.1099/mgen.0.001262 Uncited Supplementary Material 1.

10.1099/mgen.0.001262 Uncited Supplementary Material 2.

## Abbreviations

AMO: amoxicillin
AMR: antimicrobial resistance
ARC: additive uncorrelated relaxed clock
CFT: ceftriaxone
CFX: cefuroxime
GPSC: global pneumococcal sequence cluster
GTR: general time-reversible
IPD: invasive pneumococcal disease
MER: meropenem
NVT: non-vaccine type
PCV: pneumococcal conjugate vaccine
PEN: penicillin
RR: relative risk
TAX: cefotaxime
VT: vaccine type
WGS: whole-genome sequencing

## References

[1] Gladstone RA, Lo SW, Lees JA, Croucher NJ, van Tonder AJ (2019). International genomic definition of pneumococcal lineages, to contextualise disease, antibiotic resistance and vaccine impact. EBioMedicine.

[2] Lo SW, Mellor K, Cohen R, Alonso AR, Belman S (2022). Emergence of a multidrug-resistant and virulent *Streptococcus pneumoniae* lineage mediates serotype replacement after PCV13: an international whole-genome sequencing study. Lancet Microbe.

[3] Weiser JN, Ferreira DM, Paton JC (2018). *Streptococcus pneumoniae*: transmission, colonization and invasion. Nat Rev Microbiol.

[4] O’Brien KL, Wolfson LJ, Watt JP, Henkle E, Deloria-Knoll M (2009). Burden of disease caused by *Streptococcus pneumoniae* in children younger than 5 years: global estimates. Lancet.

[5] Wahl B, O’Brien KL, Greenbaum A, Majumder A, Liu L (2018). Burden of *Streptococcus pneumoniae* and Haemophilus influenzae type b disease in children in the era of conjugate vaccines: global, regional, and national estimates for 2000-15. Lancet Glob Health.

[6] Ben-Shimol S, Givon-Lavi N, Leibovitz E, Raiz S, Greenberg D (2014). Near-elimination of otitis media caused by 13-valent pneumococcal conjugate vaccine (PCV) serotypes in southern Israel shortly after sequential introduction of 7-valent/13-valent PCV. Clin Infect Dis.

[7] Ben-Shimol S, Regev-Yochay G, Givon-Lavi N, van der Beek BA, Brosh-Nissimov T (2022). Dynamics of invasive pneumococcal disease in Israel in children and adults in the 13-valent pneumococcal conjugate vaccine (PCV13) era: a nationwide prospective surveillance. Clin Infect Dis.

[8] Weinberger DM, Trzciński K, Lu Y-J, Bogaert D, Brandes A (2009). Pneumococcal capsular polysaccharide structure predicts serotype prevalence. PLoS Pathog.

[9] Ganaie F, Saad JS, McGee L, van Tonder AJ, Bentley SD (2020). A new pneumococcal capsule type, 10D, is the 100th serotype and has a large *cps* fragment from an oral *Streptococcus*. mBio.

[10] Geno KA, Gilbert GL, Song JY, Skovsted IC, Klugman KP (2015). Pneumococcal capsules and their types: past, present, and future. Clin Microbiol Rev.

[11] Rokney A, Ben-Shimol S, Korenman Z, Porat N, Gorodnitzky Z (2018). Emergence of *Streptococcus pneumoniae* serotype 12F after sequential introduction of 7- and 13-valent vaccines, Israel. Emerg Infect Dis.

[12] Lo SW, Mellor K, Cohen R, Alonso AR, Belman S Emergence of a multidrug resistant and virulent *Streptococcus pneumoniae* lineage mediates serotype replacement after PCV13. Infect Dis (except HIV/AIDS).

[13] Dagan R, Ben-Shimol S, Benisty R, Regev-Yochay G, Lo SW (2021). A nationwide outbreak of invasive pneumococcal disease in Israel caused by *Streptococcus pneumoniae* serotype 2. Clin Infect Dis.

[14] Stevens KE, Sebert ME (2011). Frequent beneficial mutations during single-colony serial transfer of *Streptococcus pneumoniae*. PLoS Genet.

[15] Chaguza C, Cornick JE, Everett DB (2015). Mechanisms and impact of genetic recombination in the evolution of *Streptococcus pneumoniae*. Comput Struct Biotechnol J.

[16] Chaguza C, Senghore M, Bojang E, Gladstone RA, Lo SW (2020). Within-host microevolution of *Streptococcus pneumoniae* is rapid and adaptive during natural colonisation. Nat Commun.

[17] Brooks LRK, Mias GI (2018). *Streptococcus pneumoniae’s* virulence and host immunity: aging, diagnostics, and prevention. Front Immunol.

[18] Bentley SD, Aanensen DM, Mavroidi A, Saunders D, Rabbinowitsch E (2006). Genetic analysis of the capsular biosynthetic locus from all 90 pneumococcal serotypes. PLoS Genet.

[19] Ganaie F, Maruhn K, Li C, Porambo RJ, Elverdal PL (2021). Structural, genetic, and serological elucidation of *Streptococcus pneumoniae* serogroup 24 serotypes: discovery of a new serotype, 24C, with a variable capsule structure. J Clin Microbiol.

[20] Lees JA, Harris SR, Tonkin-Hill G, Gladstone RA, Lo SW (2019). Fast and flexible bacterial genomic epidemiology with PopPUNK. Genome Res.

[21] Gladstone RA, Lo SW, Goater R, Yeats C, Taylor B (2020). Visualizing variation within global pneumococcal sequence clusters (GPSCs) and country population snapshots to contextualize pneumococcal isolates. Microb Genom.

[22] Belman S, Lefrancq N, Nzenze S, Downs S, du Plessis M (2023). Geographic migration and vaccine-induced fitness changes of *Streptococcus pneumoniae*. bioRxiv.

[23] Page AJ, De Silva N, Hunt M, Quail MA, Parkhill J (2016). Robust high-throughput prokaryote *de novo* assembly and improvement pipeline for Illumina data. Microb Genom.

[24] Epping L, van Tonder AJ, Gladstone RA, Bentley SD, The global pneumococcal sequencing Consortium (2018). SeroBA: rapid high-throughput serotyping of *Streptococcus pneumoniae* from whole genome sequence data. Microb Genom.

[25] Metcalf BJ, Gertz RE, Gladstone RA, Walker H, Sherwood LK (2016). Strain features and distributions in pneumococci from children with invasive disease before and after 13-valent conjugate vaccine implementation in the USA. Clin Microbiol Infect.

[26] Li Y, Metcalf BJ, Chochua S, Li Z, Gertz RE (2017). Validation of β-lactam minimum inhibitory concentration predictions for pneumococcal isolates with newly encountered penicillin binding protein (PBP) sequences. BMC Genom.

[27] Assefa S, Keane TM, Otto TD, Newbold C, Berriman M (2009). ABACAS: algorithm-based automatic contiguation of assembled sequences. Bioinformatics.

[28] Li H, Durbin R (2009). Fast and accurate short read alignment with Burrows-Wheeler transform. Bioinformatics.

[29] Li H, Handsaker B, Wysoker A, Fennell T, Ruan J (2009). The sequence alignment/map format and SAMtools. Bioinformatics.

[30] Croucher NJ, Page AJ, Connor TR, Delaney AJ, Keane JA (2015). Rapid phylogenetic analysis of large samples of recombinant bacterial whole genome sequences using Gubbins. Nucleic Acids Res.

[31] Stamatakis A (2014). RAxML version 8: a tool for phylogenetic analysis and post-analysis of large phylogenies. Bioinformatics.

[32] Didelot X, Croucher NJ, Bentley SD, Harris SR, Wilson DJ (2018). Bayesian inference of ancestral dates on bacterial phylogenetic trees. Nucleic Acids Res.

[33] Didelot X, Siveroni I, Volz EM (2021). Additive uncorrelated relaxed clock models for the dating of genomic epidemiology phylogenies. Mol Biol Evol.

[34] Rebhun U, Brown D (2015). Patterns and selectivities of urban/rural migration in Israel. DemRes.

[35] Arnon S, Shamai S (2010). Community life as a motive for migration from the urban center to the rural periphery in Israel. J Commun Psychol.

[36] Evans M (2007). An Institutional Framework for Policymaking: Planning and Population Dispersal in Israel.

[37] Sharaby N, Shiftan Y (2012). The impact of fare integration on travel behavior and transit ridership. Transport Policy.

[38] International Roadways Country Comparison. https://web.archive.org/web/20210108213930/https://www.cia.gov/the-world-factbook/field/roadways/country-comparison.

[39] Freid D (2019). Planning children’s day care centers in Israel in a changing reality. https://en.urbanclinic.huji.ac.il/sites/default/files/urbanclinic/files/planning_childrens_day_care_centers_in_israel_in_a_changing_reality_2019.pdf.

[40] Rocabert C, Fenet S, Kaufmann B, Gippet JMW (2024). Accounting for the topology of road networks to better explain human‐mediated dispersal in terrestrial landscapes. Ecography.

[41] United Nations (2021). International migration report 2019.

[42] Hong SL, Lemey P, Suchard MA, Baele G (2021). Bayesian phylogeographic analysis incorporating predictors and individual travel histories in BEAST. Curr Protoc.

