# Supplementary material for: Estimating geographical spread of Streptococcus pneumoniae within Israel using genomic data
Source: Microb Genom. 2024 Jun 24;10(6):001262. doi: 10.1099/mgen.0.001262 (PMC11261897; doi:10.1099/mgen.0.001262)
Supplement: Uncited Supplementary Material 1. [file mgen-10-01262-s001.pdf]

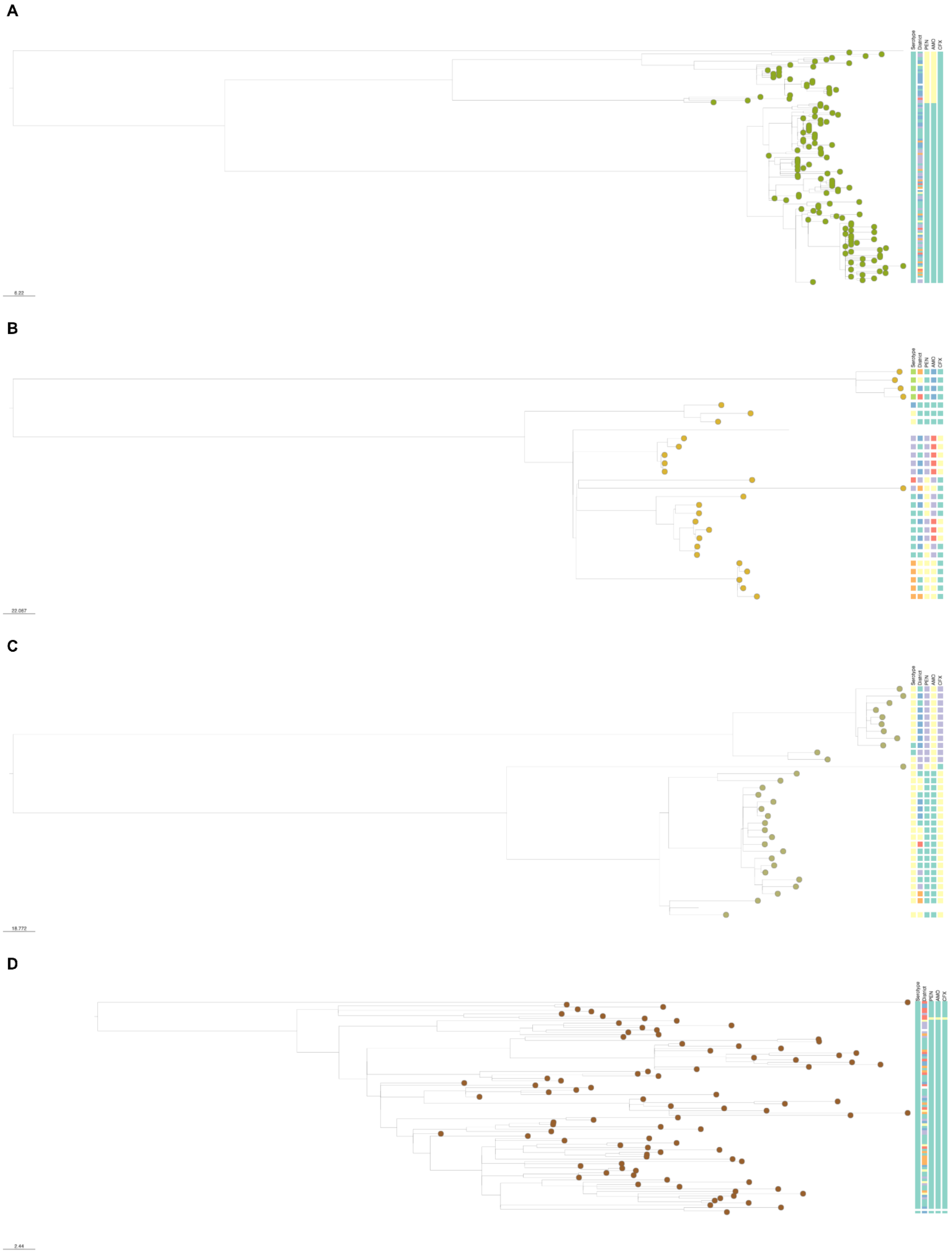

Supplementary Figure 1. the Dominant Lineages in Israel included in the study
The recombination-corrected phylogenetic tree of isolates in each lineage and their serotype, district, and AMR profiles (A) GPSC8 (B) GPSC10 (C) GPSC47 (D) GPSC55

A.

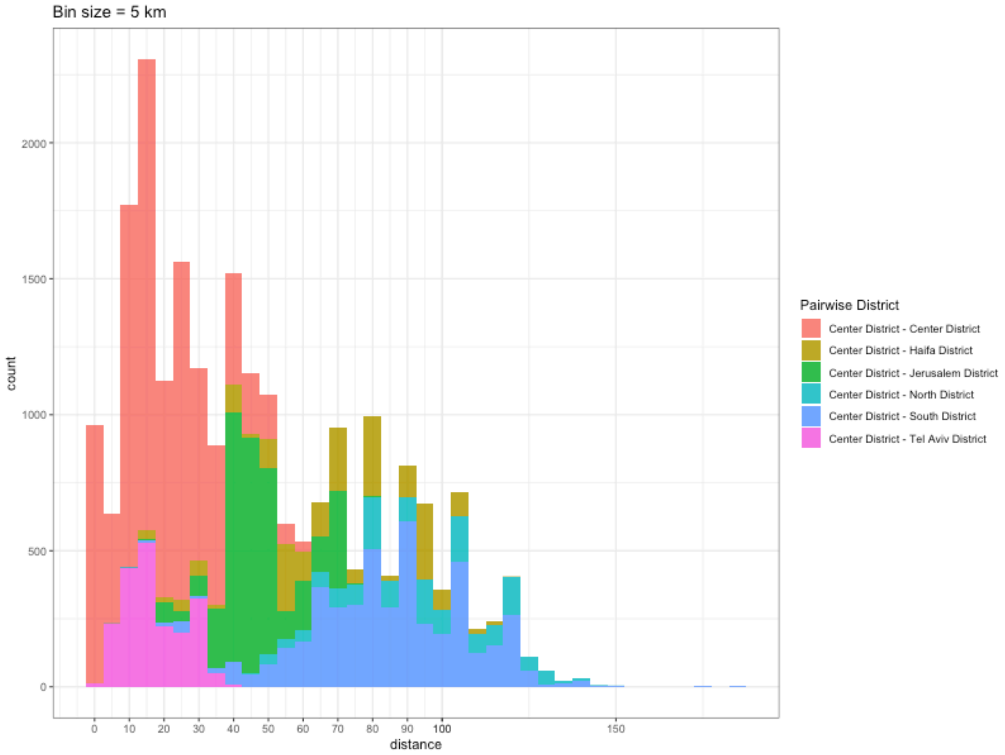
Bin size = 5 km
count
distance
Pairwise District
Center District - Center District
Center District - Haifa District
Center District - Jerusalem District
Center District - North District
Center District - South District
Center District - Tel Aviv District

B.

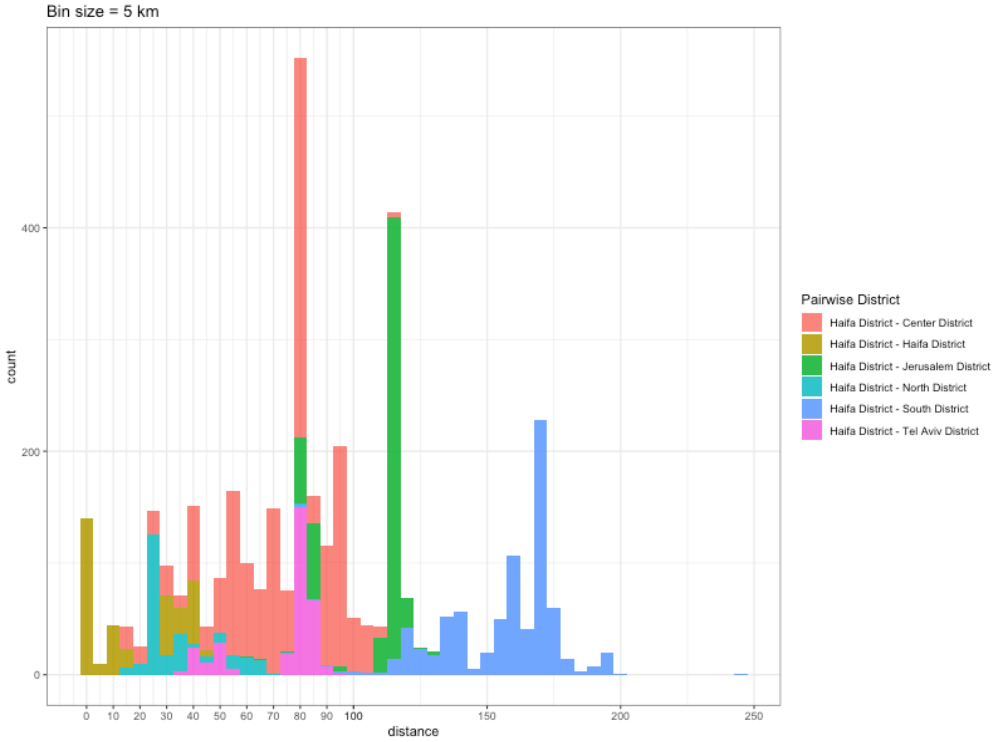
Bin size = 5 km
count
distance
Pairwise District
Haifa District - Center District
Haifa District - Haifa District
Haifa District - Jerusalem District
Haifa District - North District
Haifa District - South District
Haifa District - Tel Aviv District

C.

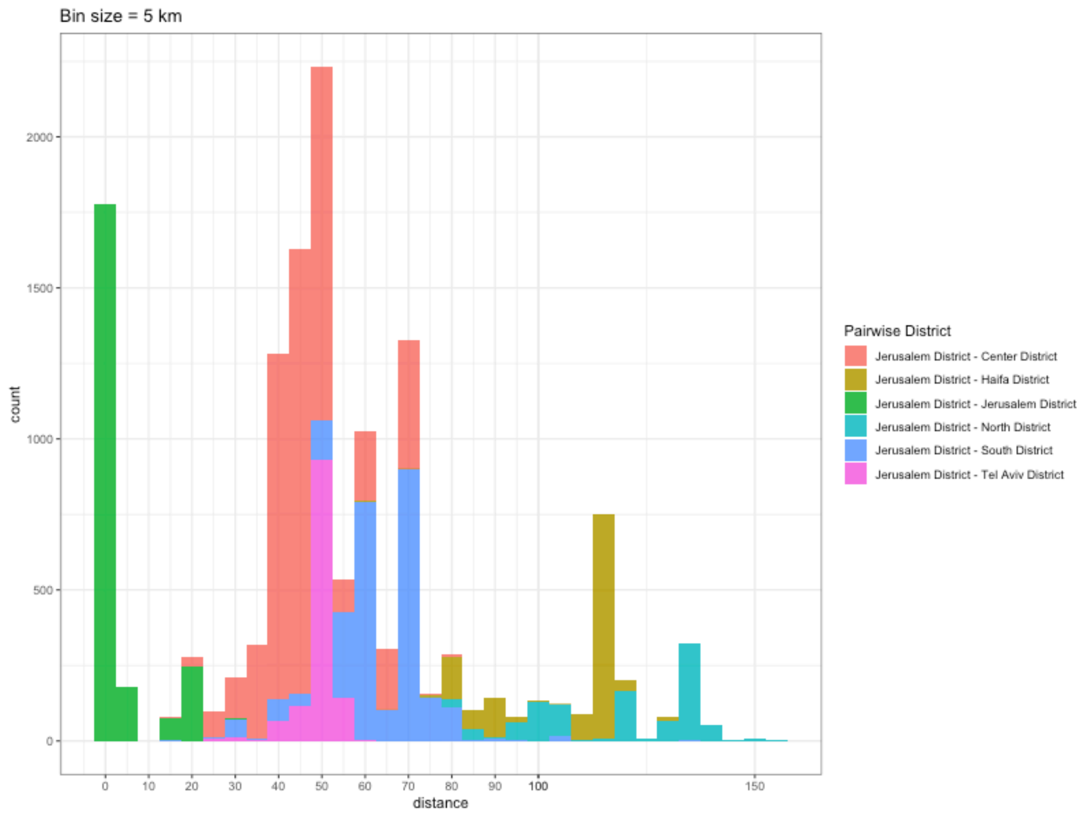

Bin size = 5 km
count
distance
Pairwise District
Jerusalem District - Center District
Jerusalem District - Haifa District
Jerusalem District - Jerusalem District
Jerusalem District - North District
Jerusalem District - South District
Jerusalem District - Tel Aviv District

D.

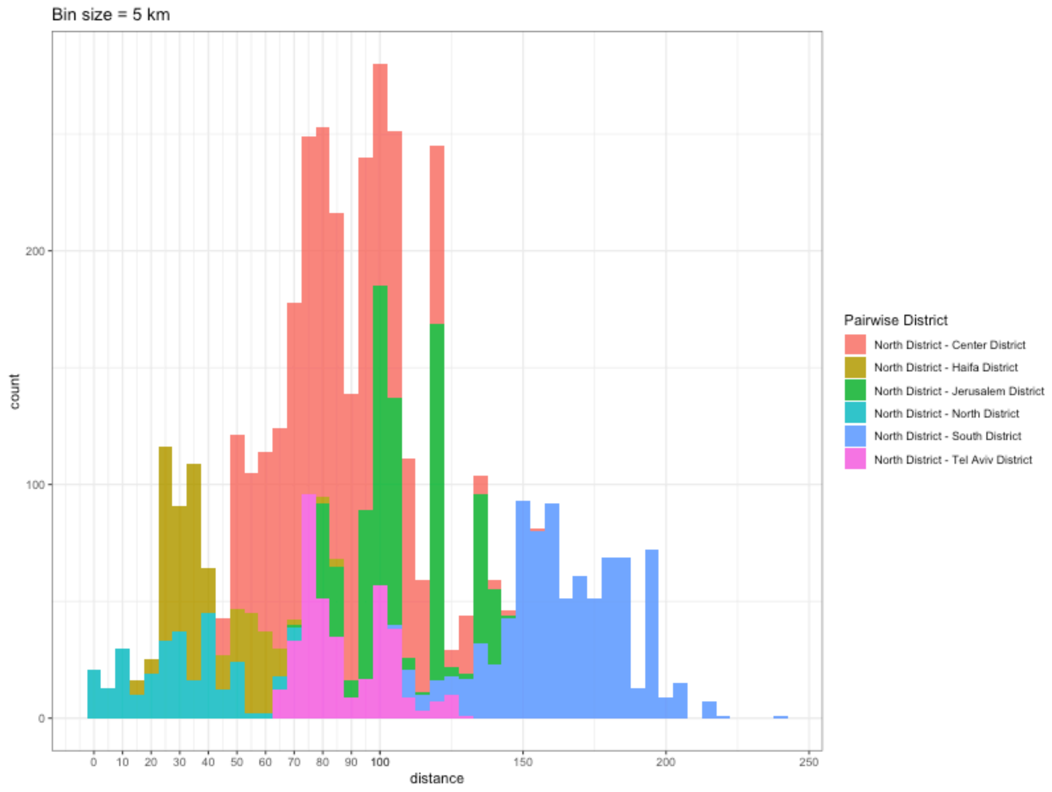

Bin size = 5 km
count
distance
Pairwise District
North District - Center District
North District - Haifa District
North District - Jerusalem District
North District - North District
North District - South District
North District - Tel Aviv District

E.

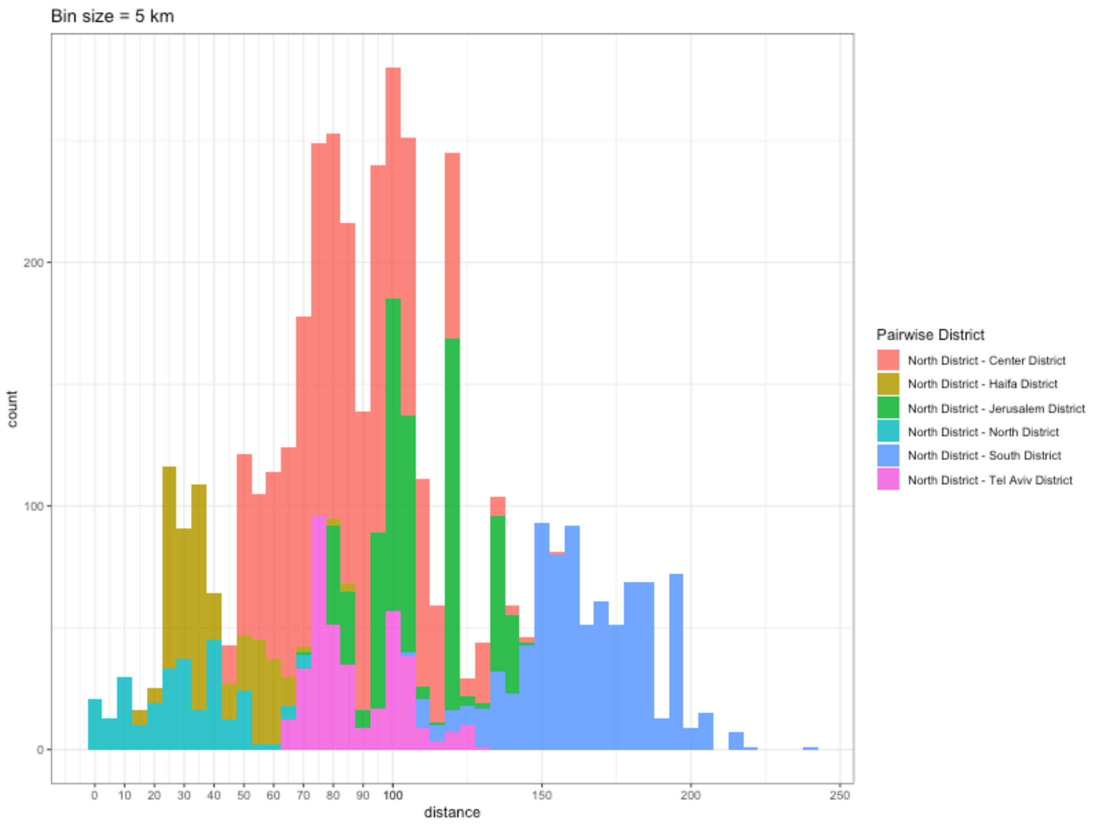

F.

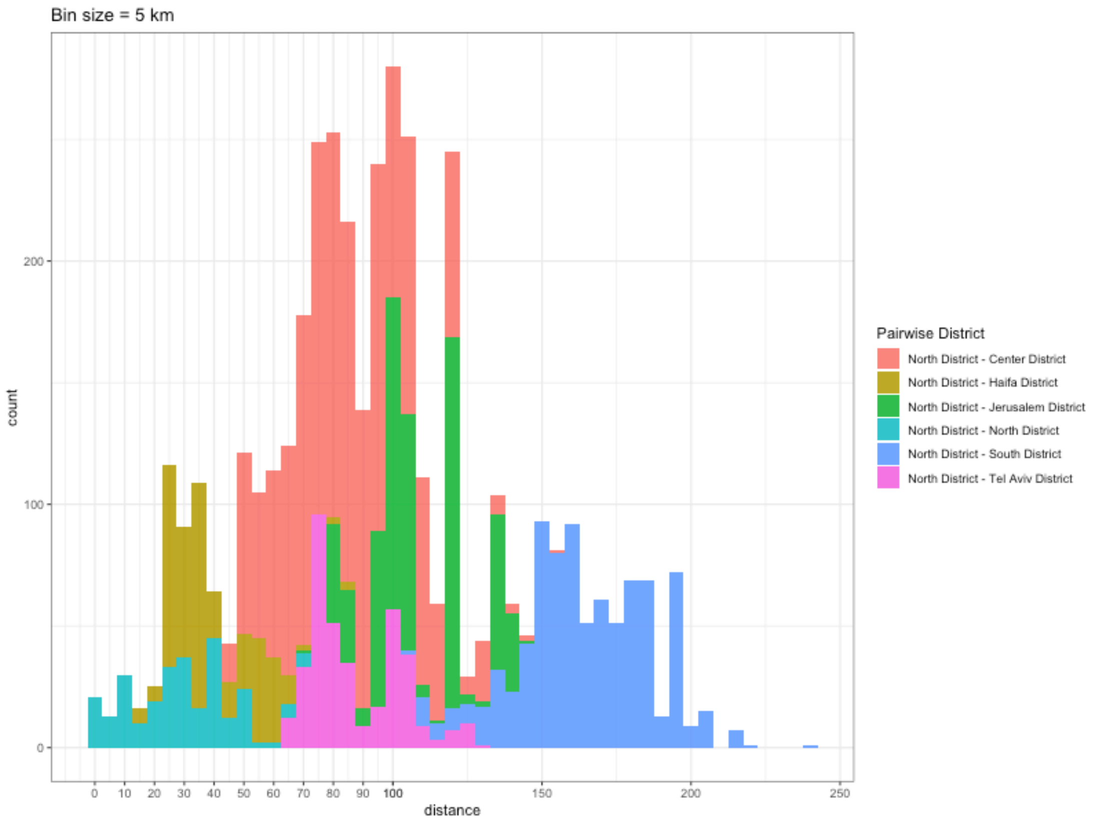

Supplementary Figure 2. Pairwise distance distribution of collected isolates in Dominant Lineages across Israel. The colours of the bars indicate the districts of each pair of isolates. The bin size of the histogram is 5 km. (A) Pairwise distance distribution with at least one isolate collected from Central district (B) Pairwise distance distribution with at least one isolate collected from Haifa district (C)

Pairwise distance distribution with at least one isolate collected from Jerusalem district (D) Pairwise distance distribution with at least one isolate collected from North district (E) Pairwise distance distribution with at least one isolate collected from South district (F) Pairwise distance distribution with at least one isolate collected from Tel Aviv district

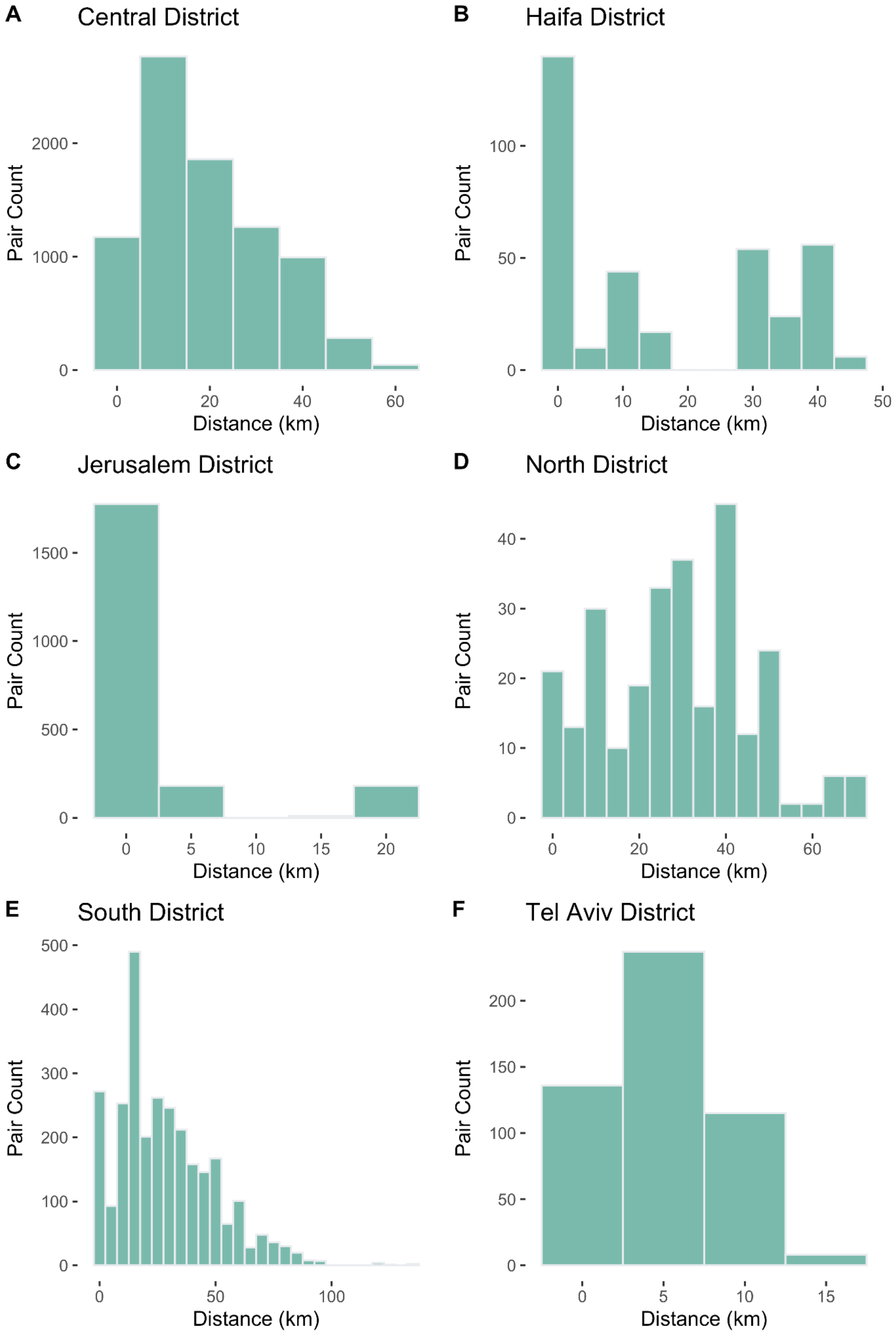

Supplementary Figure 3. Pairwise distance of collected isolates in Dominant Lineages within each district in Israel. The bin size of the histogram is 5 km.

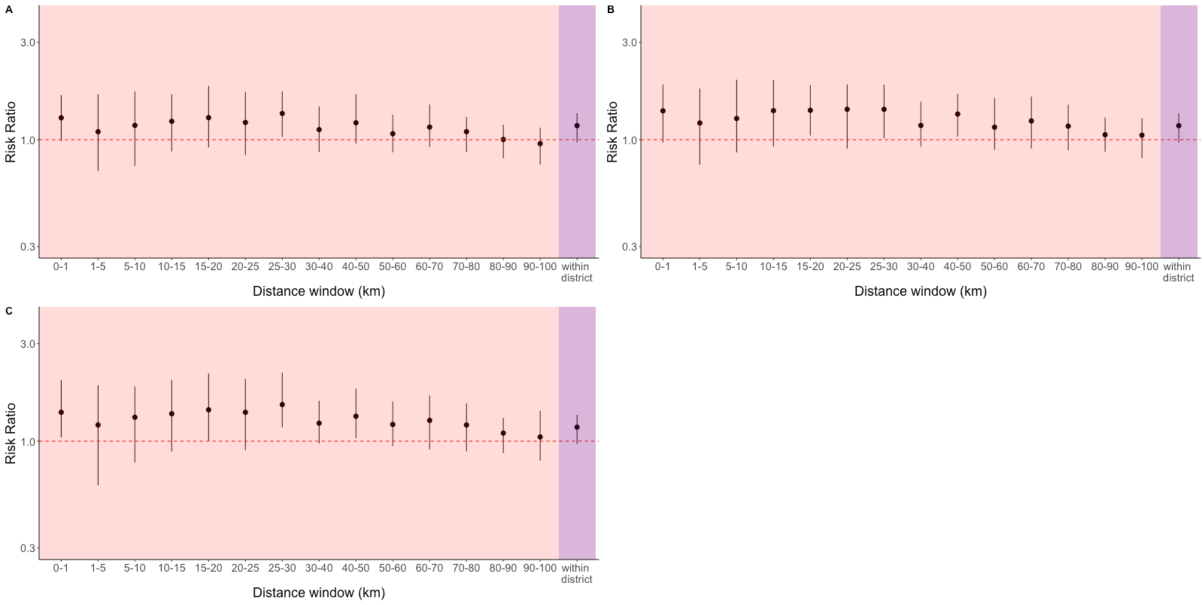

Supplementary Figure 4. Sensitivity analysis of the geographic structure at lineage level. Relative risk ratio of spread at lineage level was calculated using Israeli disease isolates (N=1159). (Purple) Relative risk ratio of spread at lineage level when comparing pairs of isolates within districts to between districts. (Pink) Relative risk ratio of spread at lineage level when comparing pairs of isolates within the rolling distance windows to pairs of isolates (A) >50 km, (B) >80 km and (C) > 100 km apart. Red dashed line highlights a relative ratio of 1, representing a significant difference in spread. The dots and lines represent 2.5, 50, and 97.5 percentiles of the confidence intervals.

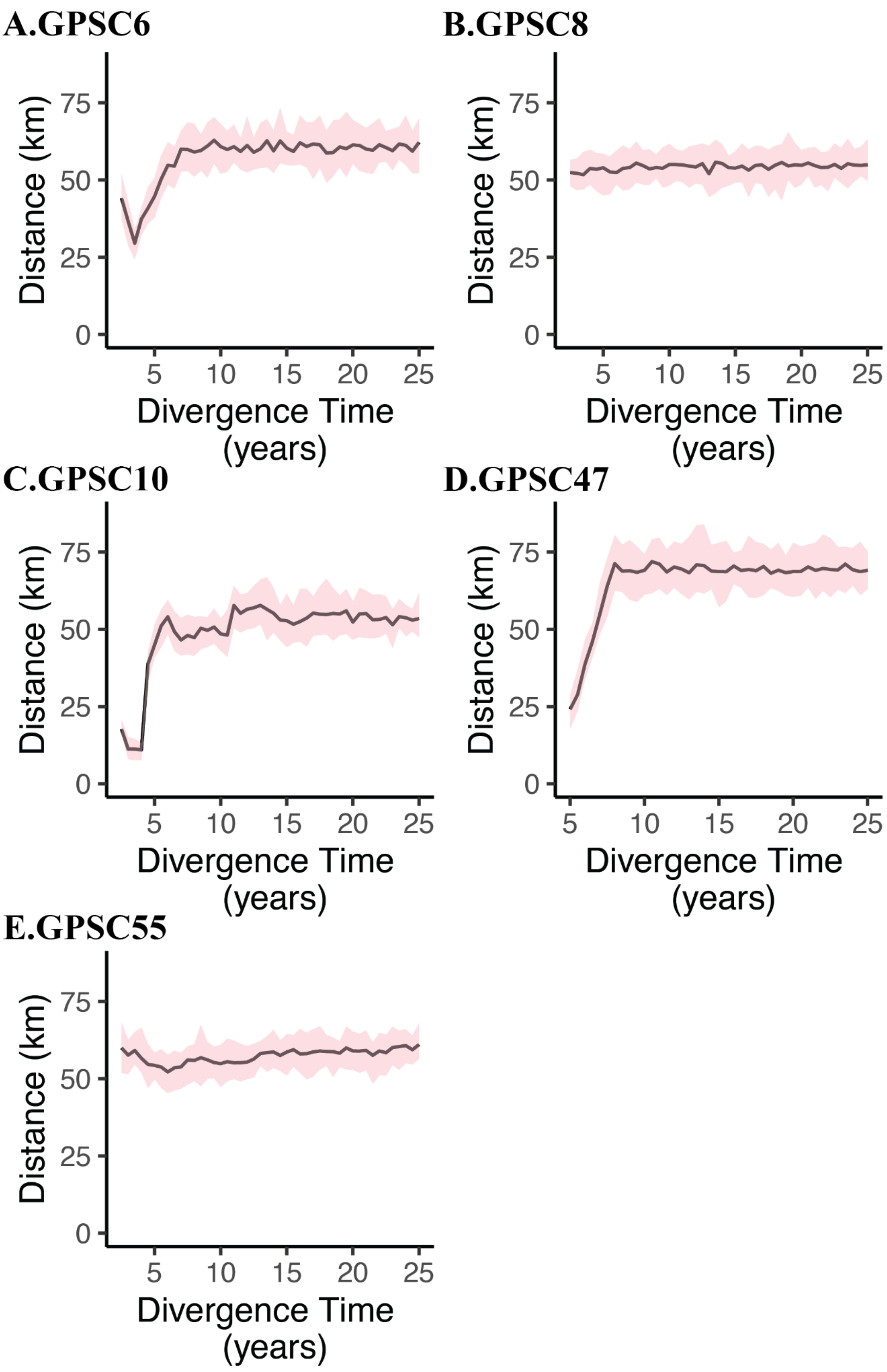

Supplementary Figure 5. The relationship between pairwise cumulative divergence time and mean geographic distance with 95% confidence interval (red) within each GPSC in Israel.

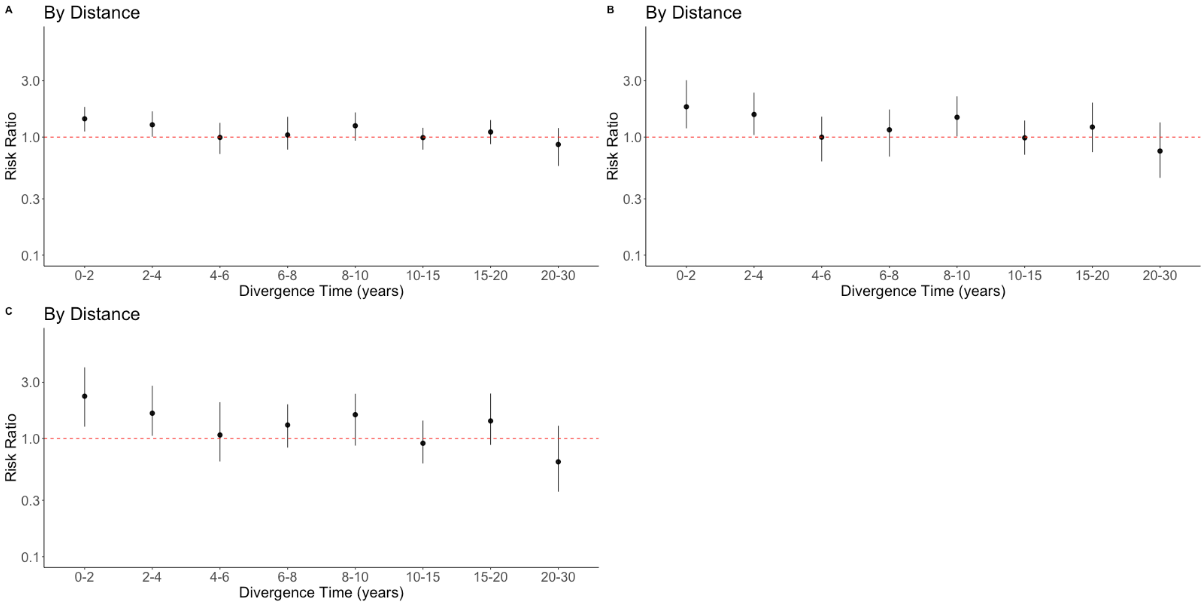

Supplementary Figure 6. Sensitivity analysis on estimation of the homogenisation time across Israel The relationship between pairwise divergence time and relative risk ratio was investigated using 5 Dominant GPSCs in the study (N=361). The relationship between pairwise divergence time and relative risk ratio when comparing pairs of isolates within 30 km to pairs of isolates (A) > 0 km, (B) > 50 and (C) >80 km apart. Red dashed line highlights a relative ratio of 1, representing a significant difference in spread. The dots and lines represent 2.5, 50, and 97.5 percentiles of the confidence intervals.

A

Rate=8.88e+00,MRCA=1998.25,R2=0.33,p<1.00e−04

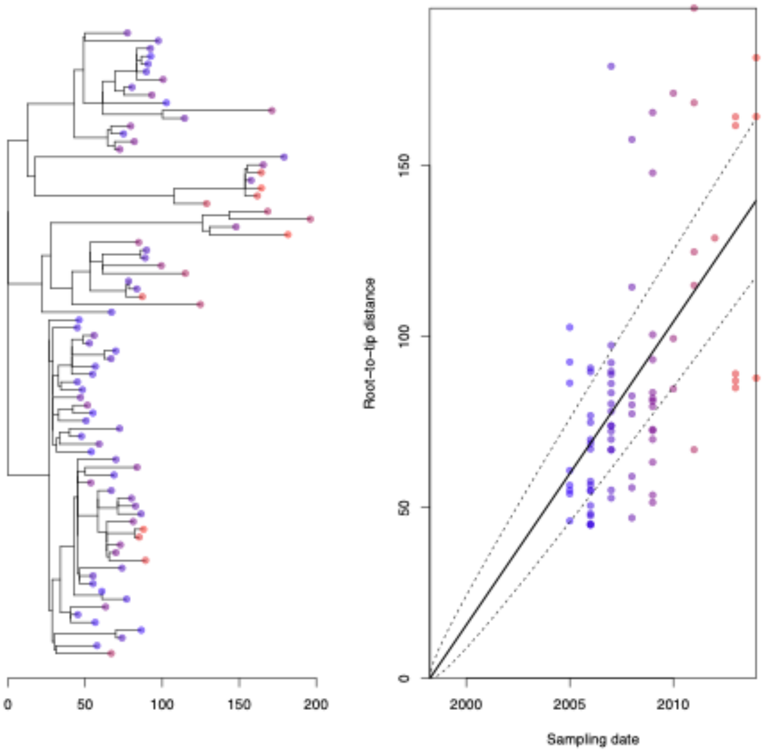

B

Rate=1.64e+00,MRCA=1940.12,R2=0.38,p<1.00e−04

GPSC8

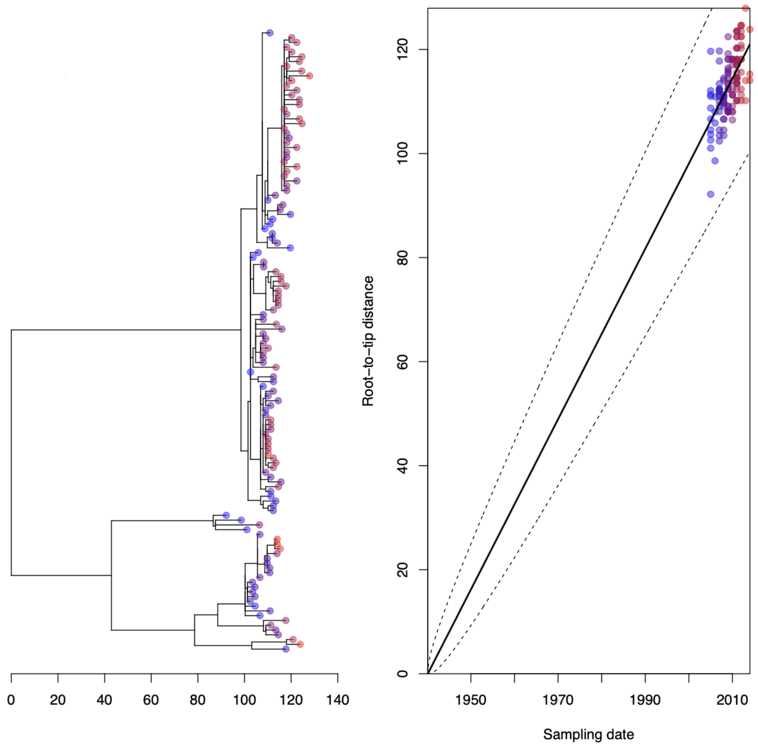

C

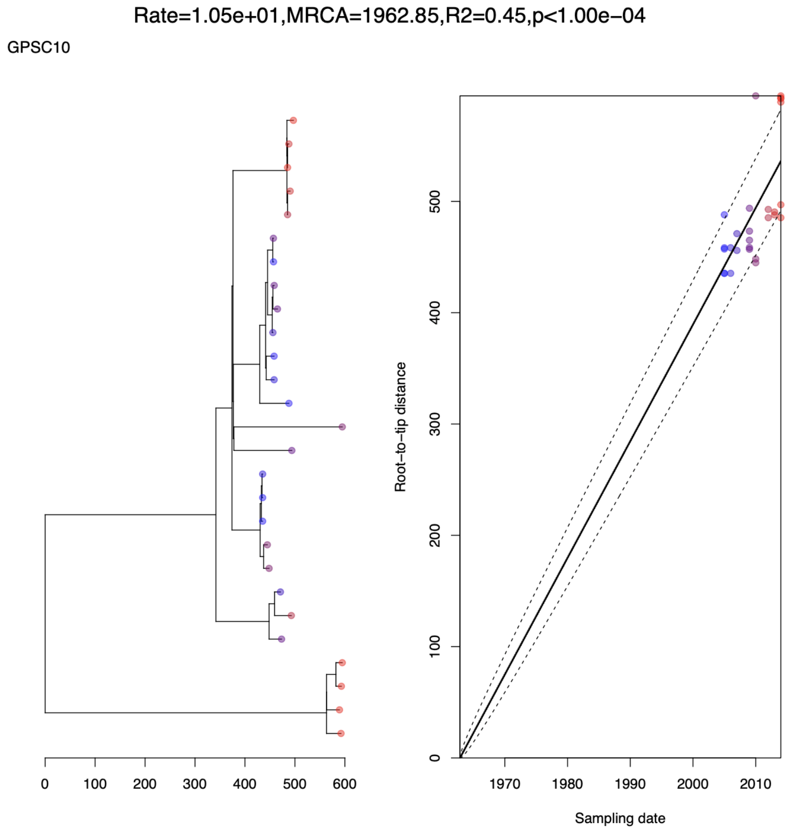
Rate=1.05e+01,MRCA=1962.85,R2=0.45,p<1.00e−04
GPSC10
Root−to−tip distance
Sampling date

D

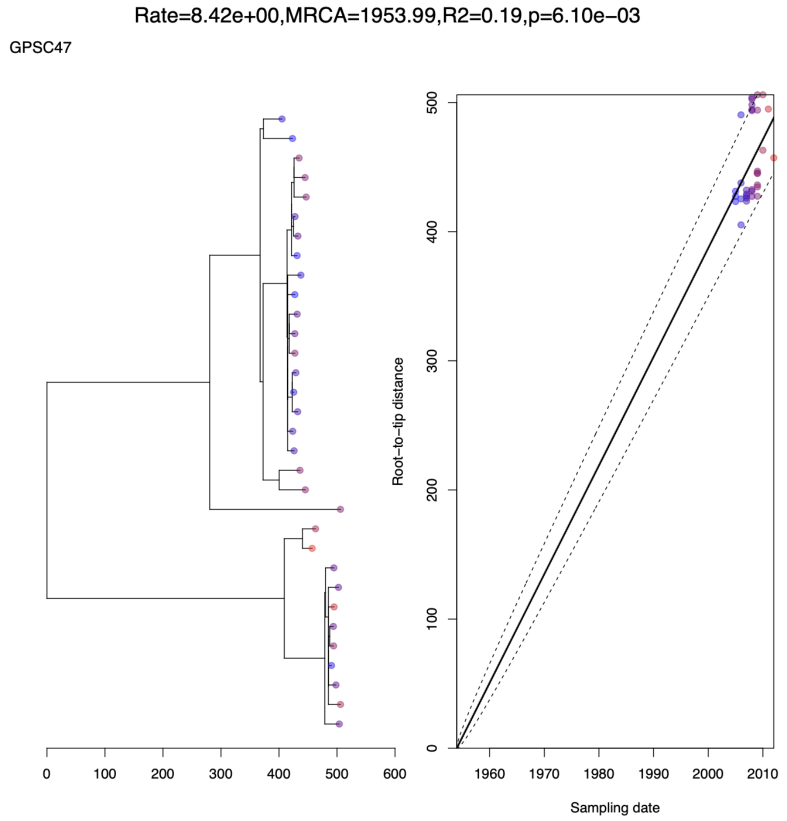
Rate=8.42e+00,MRCA=1953.99,R2=0.19,p=6.10e−03
GPSC47
Root−to−tip distance
Sampling date

E

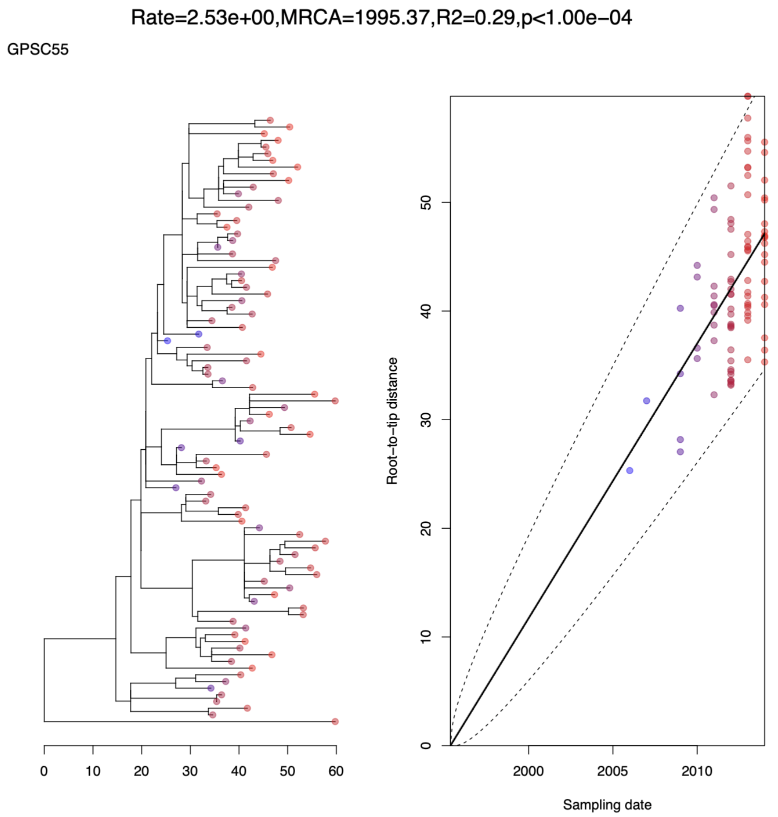

Supplementary Figure 7. Time-resolved phylogenies for dominant GPSC lineages (A) GPSC6 (B) GPSC8 (C) GPSC10 (D) GPSC47 and (E) GPSC55, in Israel *S. pneumoniae* population.

A

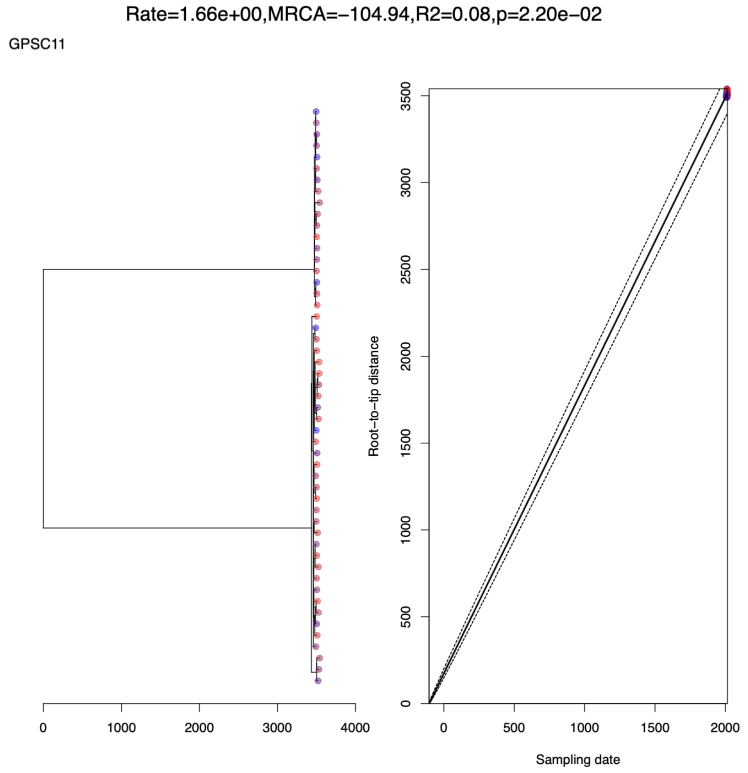

B

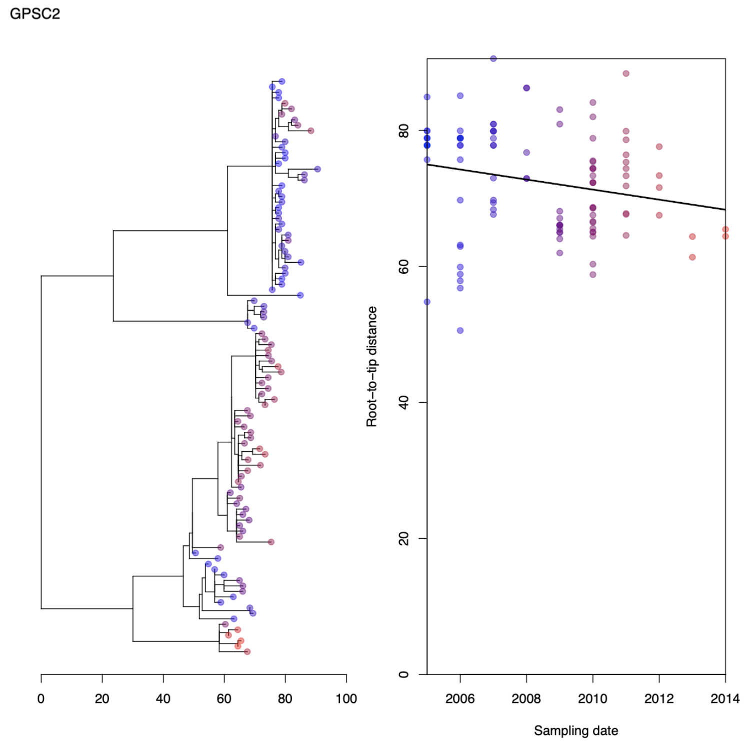

Supplementary Figure 8. GPSC lineages that are excluded due to erroneous estimation of divergence time. (A) Recent clonal expansion or distinct clades within GPSC11 lineage caused erroneous estimates of MRCA. Similar events have been observed in GPSC13 and GPSC16. (B) Negative correlation between sampling date and root-to-tip distance was shown in GPSC2. Similar events have been observed in GPSC5, GPSC7, GPSC12, GPSC31

A

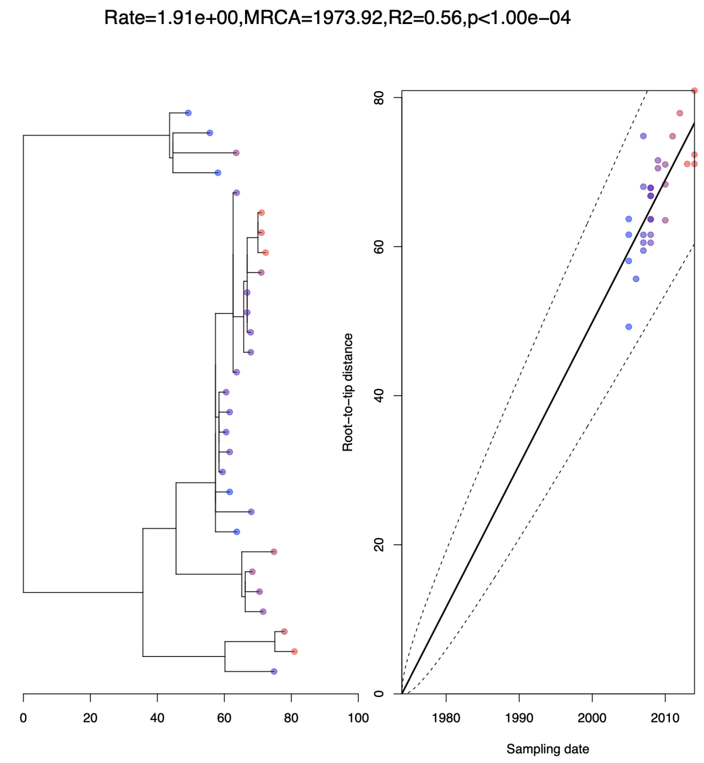

B

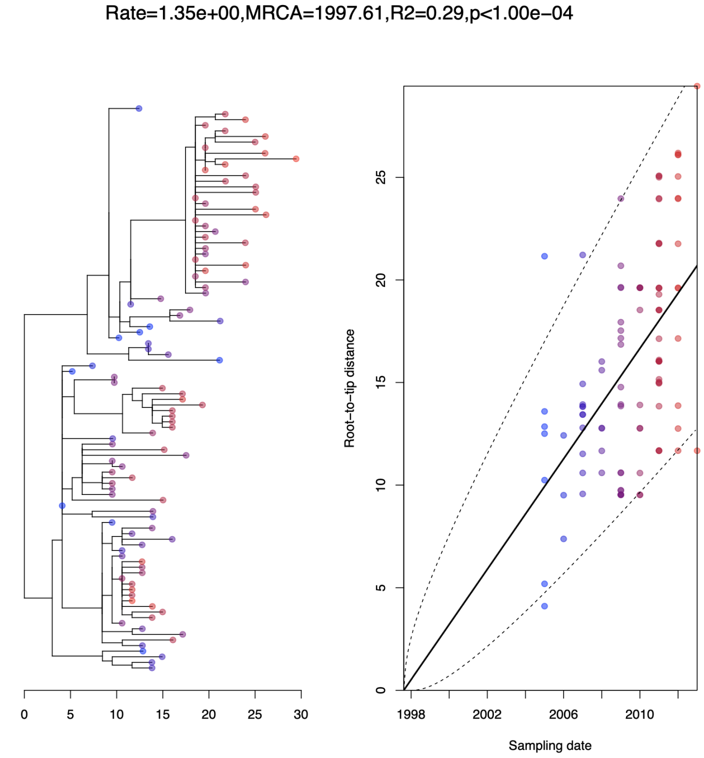

Supplementary Figure 9. GPSC8 was split into two subclades (A and B) to improve the divergence time estimation

GPSC	Center	Haifa	Jerusalem	North	South	Tel Aviv
6	36	7	15	6	13	4
8	43	5	33	5	33	11
10	10	3	1	1	9	3
47	9	5	5	1	10	2
55	34	7	14	11	13	12
Others	259	66	147	48	235	58
Total	391	93	215	72	313	90

Supplementary Table 1. The number of samples collected in each district for each Dominant GPSC and other GPSCs.
